# Outbreaks of Unexplained Neurologic Illness — Muzaffarpur, India, 2013–2014

**Published:** 2015-01-30

**Authors:** Aakash Shrivastava, Padmini Srikantiah, Anil Kumar, Gyan Bhushan, Kapil Goel, Satish Kumar, Tripurari Kumar, Raju Mohankumar, Rajesh Pandey, Parvez Pathan, Yogita Tulsian, Mohan Pappanna, Achhelal Pasi, Arghya Pradhan, Pankaj Singh, D. Somashekar, Anoop Velayudhan, Rajesh Yadav, Mala Chhabra, Veena Mittal, Shashi Khare, James J Sejvar, Mayank Dwivedi, Kayla Laserson, Kenneth C. Earhart, P. Sivaperumal, A. Ramesh Kumar, Amit Chakrabarti, Jerry Thomas, Joshua Schier, Ram Singh, Ravi Shankar Singh, A.C. Dhariwal, L.S. Chauhan

**Affiliations:** 1 National Centre for Disease Control, Directorate General of Health Services, Ministry of Health and Family Welfare, Government of India, New Delhi, India; 2 Global Disease Detection Program, CDC, New Delhi, India; 3 Division of Global Health Protection, Center for Global Health, CDC; 4 Muzaffarpur District Health Department, Government of Bihar, Muzaffarpur, India; 5 India Epidemic Intelligence Service Cohort 1, National Centre for Disease Control, New Delhi, India; 6 India Epidemic Intelligence Service Cohort 2, National Centre for Disease Control, New Delhi, India; 7 National Center for Enteric and Zoonotic Diseases, CDC; 8 National Institute of Occupational Health, Indian Council of Medical Research, Ahmedabad, India; 9 National Center for Environmental Health, CDC; 10 National Vector Borne Disease Control Programme, Directorate General of Health Services, Ministry of Health and Family Welfare, Government of India, New Delhi, India

Outbreaks of an unexplained acute neurologic illness affecting young children and associated with high case-fatality rates have been reported in the Muzaffarpur district of Bihar state in India since 1995. The outbreaks generally peak in June and decline weeks later with the onset of monsoon rains. There have been multiple epidemiologic and laboratory investigations of this syndrome, leading to a wide spectrum of proposed causes for the illness, including infectious encephalitis and exposure to pesticides. An association between illness and litchi fruit has been postulated because Muzaffarpur is a litchi fruit–producing region (Figure 1). To better characterize clinical and epidemiologic features of the illness that might suggest its cause and how it can be prevented, the Indian National Centre for Disease Control (NCDC) and CDC investigated outbreaks in 2013 and 2014. Clinical and laboratory findings in 2013 suggested a noninflammatory encephalopathy, possibly caused by a toxin. A common laboratory finding was low blood glucose (<70 mg/dL) on admission, a finding associated with a poorer outcome; 44% of all cases were fatal. An ongoing 2014 investigation has found no evidence of any infectious etiology and supports the possibility that exposure to a toxin might be the cause. The outbreak period coincides with the month-long litchi harvesting season in Muzaffarpur. Although a specific etiology has not yet been determined, the 2014 investigation has identified the illness as a hypoglycemic encephalopathy and confirmed the importance of ongoing laboratory evaluation of environmental toxins to identify a potential causative agent, including markers for methylenecyclopropylglycine (MCPG), a compound found in litchi seeds known to cause hypoglycemia in animal studies (1*–*3). Current public health recommendations are focused on reducing mortality by urging affected families to seek prompt medical care, and ensuring rapid assessment and correction of hypoglycemia in ill children.

## 2013 Outbreak Investigation

During May 17–July 22, 2013, a total of 133 children were admitted to the two main referral hospitals in Muzaffarpur with illnesses that met the investigation case definition of acute onset seizures or altered mental status within 7 days of admission in a child aged <15 years. Of these, 94 (71%) patients were from Muzaffarpur; other patients were from six neighboring districts. Among the 133 patients, 71% were aged 1–5 years, 94% had generalized seizures, and 93% had altered mental status. Most (61%) were afebrile at admission; the case fatality rate was 44%. Among 56 patients with cerebrospinal fluid (CSF) examined, 31 (55%) had normal cytology (white blood cell [WBC] count = <5/mm^3^); 48 of 59 (81%) had CSF normal protein (<45 mg/dL), and 46 of 61 (75%) had normal CSF glucose (>45 mg/dL) levels. At admission, 20 (21%) of 94 patients had hypoglycemia (blood glucose <70 mg/dL).

CSF samples were tested at NCDC for selected infectious pathogens known to cause encephalitis in the region. Of 60 CSF specimens tested for Japanese encephalitis virus by immunoglobulin M (IgM) capture enzyme-linked immunosorbent assay, 33 by polymerase chain reaction, and 33 by virus isolation, all were negative. Sixteen convalescent serum specimens, collected 14 days after illness onset, also were negative for Japanese encephalitis virus by IgM assay. Thirty CSF specimens examined by reverse transcription–polymerase chain reaction for flaviviruses and 13 examined more specifically for West Nile virus also were negative, as were 23 evaluated for Chandipura virus. Fourteen CSF specimens evaluated by polymerase chain reaction and virus isolation for enteroviruses did not demonstrate evidence of infection.

Analysis of risk factors for death among 94 affected children showed that low blood glucose at admission was more common among those who died (odds ratio = 2.6; 95% confidence interval [CI] = 1.0–7.2). A case-control study enrolled 101 case-patients and 202 age-matched controls, 101 from the hospital and 101 from the community. Ill children had spent a greater amount of time in agricultural fields or orchards (matched odds ratio = 2.6; CI = 1.2–5.2) than controls. Anthropometric data on 24 patents suggested that younger patients (those aged <5 years) were more likely to have wasting (>2 standard deviations below the median weight for height of the reference population) than controls in the same age group (p = 0.03).

Data collected during the 2013 investigation suggested that the illness was more likely to be a noninflammatory encephalopathy than an infectious encephalitis, and raised concern for the possibility of a toxin-mediated illness. Although the 2013 investigation did not identify a specific etiology, key recommendations shared with state and district health officials focused on reduction of mortality, including provision of glucometers for hospitals and peripheral health facilities and rapid assessment and treatment of hypoglycemia in children with suspected illness.

## 2014 Outbreak Investigation

Building on the 2013 findings, NCDC and CDC again investigated this syndrome in 2014, using 1) facility-based clinical surveillance, 2) epidemiologic case-control and environmental studies to examine risk factors for illness, including toxin exposures and nutritional indices, and 3) comprehensive laboratory evaluation of patient specimens and environmental samples to search for infectious pathogens as well as selected pesticides, heavy metals, and naturally occurring plant or fruit toxins. Suspected patients were promptly tested for hypoglycemia on arrival at the hospital, before being given any treatment. Patients admitted with the suspected outbreak illness were recommended to receive immediate intravenous dextrose therapy.

**FIGURE 2:**
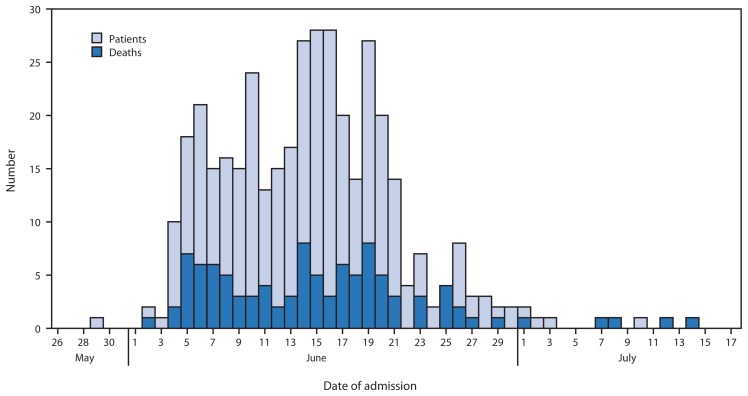
Number of patients admitted to two referral hospitals with unexplained acute neurologic illness, by date of admission — Muzaffarpur, India, May 26–July 17, 2014

During May 26–July 17, 2014, a total of 390 patients admitted to the two referral hospitals in Muzaffarpur with illnesses that met the same case definition used in 2013 were evaluated by the NCDC/CDC investigation team. Among the patients, 213 (55%) were male, the median age was 4 years (range = 6 months–14 years), and 280 (72%) were aged 1–5 years. Most patients were from Muzaffarpur district (70%), although patients also were reported from six surrounding districts. As in previous years, clustering of cases was not observed; the illness of each affected child appeared to be an isolated case in various villages (approximate population per village = 1,000). The outbreak peaked in mid-June, with 147 cases reported during June 8–14, 2014. The number of cases declined significantly after the onset of monsoon rains on June 21, 2014 (Figure 2).

Caregivers reported that affected children were previously healthy and experienced an acute onset of convulsions, often between 4:00 a.m. and 8:00 a.m., frequently followed by a decreased level of consciousness. Of 345 patients with recorded data, 324 (94%) had seizures on admission, and 267 (77%) had altered mental status. Of 357 patients with body temperature measured on admission, 219 (61%) were afebrile (≤99.5°F [≤37.5°C]). The case-fatality rate was 31%.

Detailed clinical evaluation of 52 patients within 12 hours of admission elicited a history of generalized tonic or tonic-clonic seizures in 100%. Upper motor neuron findings of generalized hypertonia and Babinksi’s sign were observed in approximately one third of patients; focal neurologic deficits were rare. Brain magnetic resonance imaging of 16 patients selected at random revealed no focal abnormalities or changes suggestive of inflammation; eight patients (50%) showed mild to moderate cerebral edema. Electroencephalography in 30 cases demonstrated findings consistent with generalized encephalopathy in 22 (73%); seven demonstrated epileptiform discharges. Overall, neurologic findings suggested a diffuse encephalopathy with seizures and cerebral edema.

Of 62 patients with CSF collected for analysis, 52 (84%) had normal WBC counts, 58 (94%) had normal protein, and 49 (79%) had normal glucose levels. Of 327 patients with blood glucose measurement on admission, the median blood glucose level was 48 mg/dL, and 171 (52%) and 204 (62%) patients had glucose levels of ≤50 mg/dL and ≤70 mg/dL, respectively. Laboratory diagnostic testing of 17 CSF specimens for Japanese encephalitis virus and West Nile virus by polymerase chain reaction was negative. Additionally, evaluation of 12 CSF specimens with a multiplex polymerase chain reaction platform assay with the capacity to detect 11 viruses* also was negative.

### Discussion

The 2013 and 2014 Muzaffarpur investigations indicate that this outbreak illness is an acute noninflammatory encephalopathy. This is supported by clinical and laboratory findings, inclusive of negative diagnostic results for the most common pathogens that cause infectious encephalitis in this region. Laboratory data indicate that significant hypoglycemia is an important presenting feature of illness. Furthermore, the implementation of the 2013 recommendations for rapid assessment and correction of hypoglycemia might, in part, have helped to reduce mortality (44% in 2013 versus 31% in 2014).

Although the underlying cause of this illness remains unknown, initial clinical and laboratory results of the 2014 investigation confirm the importance of systematically evaluating toxins and agents with the potential to cause acute encephalopathy. Furthermore, the consistent finding of hypoglycemia among affected children underscores the importance of examining the possible role of compounds that might acutely result in low blood sugar, seizures, and encephalopathy, including the possible role of MCPG in litchis. Outbreaks of similar acute neurologic illnesses occurring in litchi-growing regions of Bangladesh and Vietnam have been reported (4*,*5) raising further interest in a possible association between litchis and this illness. The investigation in Bangladesh focused primarily on the possibility that pesticides used seasonally in litchi orchards might be involved, but no specific pesticide was implicated. The investigation in Vietnam focused primarily on possible infectious agents that might be present seasonally near litchi fruit plantations but found none to explain the outbreak. In Muzaffarpur, MCPG is hypothesized to cause acute hypoglycemia and illness through a similar mechanism to hypoglycin A, a toxin that has been reported to cause acute encephalopathy in the West Indies and West Africa after consumption of unripe ackee, a fruit in the same botanical family as litchi (6*–*9).

**What is already known on this topic?**

Seasonal outbreaks of an unexplained acute neurologic illness affecting young children and associated with high case fatality have been reported from Muzaffarpur, India, since 1995. Multiple potential etiologies have been proposed, including infectious encephalitis and pesticide exposure, but not systematically assessed.

**What is added by this report?**

Outbreak investigations in 2013 and 2014 helped to classify this illness as a noninflammatory encephalopathy. Approximately 60% of patients had low blood glucose (<70 mg/dL) on admission, which was associated with poorer outcomes and prompted recommendations for rapid assessment and treatment of low blood glucose. The low blood glucose raised the possibility that exposure to a toxin could result in low blood glucose, seizures, and encephalopathy. One specific hypothesis was that exposure to MCPG, a toxin in litchis, might cause acute hypoglycemia and encephalopathy in some children. Laboratory investigations to assess this possibility and understand why only some children are affected are ongoing.

**What are the implications for public health practice?**

A collaborative, multidisciplinary systematic investigation of this outbreak has been essential to correctly classify this illness and focus analytic efforts on evaluation of testable data-driven hypotheses to identify a potential etiology. The implementation of the 2013 recommendations for rapid assessment and correction of hypoglycemia might, in part, have helped to reduce mortality (44% in 2013 compared with 31% in 2014). Public health recommendations are focused on advising affected families to seek prompt medical attention, and advising healthcare providers to rapidly assess and correct hypoglycemia in ill children.

As part of the collaborative investigation, blood and urine specimens of affected children are being systematically assayed by the Indian National Institute for Occupational Health and CDC for pesticide metabolites, heavy metals, and markers for MCPG and its metabolites. Litchi fruits collected from orchards that border the homes of affected children are being examined for MCPG markers, and environmental samples (local vegetation, food grains, and water) collected from homes of patients and controls are being evaluated for pesticide residues. Additionally, analysis of epidemiologic data collected in the 2014 case-control study, including detailed histories regarding consumption of litchis or exposure to pesticides, might elucidate potential risk factors for illness among these children.

Analysis of nutritional indices and other host factors is planned to search for an explanation for the lack of clustering of cases in these outbreaks. Until an etiology for this illness is identified, current public health and clinical recommendations are focused on reducing mortality by ensuring families with affected children rapidly access medical attention, and health care providers promptly assess for and correct hypoglycemia.

## Figures and Tables

**FIGURE 1:**
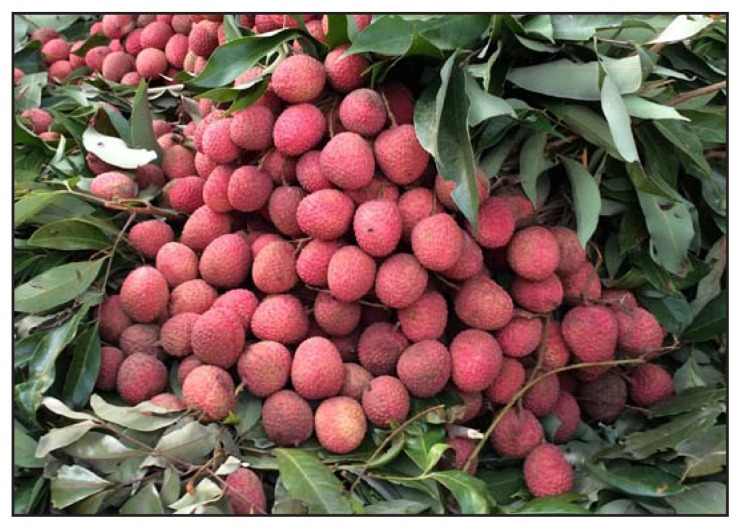
Litchi fruit orchards have been a focus of the investigation into outbreaks of unexplained neurologic illness among children — Muzaffarpur, India, 2013–2014

## Footnotes

* Herpes simplex viruses 1 and 2, human herpes viruses 6 and 7, cytomegalovirus, varicella zoster virus, Epstein-Barr virus, parechovirus, adenovirus, enteroviruses, and parvovirus B19.
